# Employing and Interpreting a Machine Learning Target-Cognizant Technique for Analysis of Unknown Signals in Multiple Reaction Monitoring

**DOI:** 10.1109/access.2021.3056955

**Published:** 2021-02-10

**Authors:** RYAN A. MCCARTHY, ANANYA SEN GUPTA

**Affiliations:** Department of Electrical and Computer Engineering, The University of Iowa, Iowa City, IA 52242, USA; Iowa Technology Institute (ITI), The University of Iowa, Iowa City, IA 52242, USA

**Keywords:** Machine learning, GC/MS/MS, PCBs, signal processing

## Abstract

The aim of this interdisciplinary work is a robust signal processing and autonomous machine learning framework to associate well-known (target) as well as any potentially unknown (non-target) peaks present within gas chromatography-mass spectrometry (GC/MS/MS) raw instrument signal. Particularly, this work evaluates three machine learning algorithms abilities to autonomously associate raw signal peaks based on accuracy in training and testing. A target is a known congener that is expected to be present within the raw instrument signal and a non-target is an unknown or unexpected compound. Autonomously identifying target peaks within the GC/MS/MS and associating them with non-target peaks can help improve the analysis of collected samples. Association of peaks refers to classifying peaks as known congeners regardless if the peak is a target or non-target. Uncertainty of peaks fitted and discovered through raw instrument signals from GC/MS/MS data is assessed to create topographical illustrations of target annotated peaks among sample raw instrument signals collected across diverse locations in the Chicago area. The term “annotated peak” is used to assign peaks found at specific retention times as a known congener. Adaptive signal processing techniques are utilized to smooth data and correct baseline drifts as well as detect and separate coeluted (overlapped) peaks in the raw instrument signal to provide key feature extraction. 150 air samples are analyzed for individual polychlorinated biphenyls (PCB) with GC/MS/MS across Chicago, IL. 80% of the data is used for training classification of target PCBs and 20% of the data is evaluated to identify and associate consistently occurring non-target peaks with target PCBs. A random forest classifier is used to associate identified peaks to target PCB peaks. Geographical topographical representations of target PCBs in the raw instrument signal demonstrates how PCBs accumulate and degrade in certain locations.

## I. INTRODUCTION

Recent autonomous target-cognizant analysis in raw signal data are increasingly used in interdisciplinary work such as environmental contamination and health sciences. Target-cognizant analysis annotates the unknown MRM measurements by comparing against the MRM measurements of known congeners. Information rich raw signals provide relevant analysis of various contributing analytes which occupy specific positions within the retention time of the instrument signal that can be interpreted through target-cognizant approaches. Though target-cognizant approaches provide expert analysis of targets, these approaches are time consuming, have confirmation biased, and exclude potential contributions of non-target information which provide more comprehensive knowledge of the raw signal data. Target, in this work, is a known congener that is present within the signal at specific retention times while a non-target is an unexpected unknowns or interferences consistently detected across multiple samples. Through machine learning techniques, an a priori target-cognizant approach is initially considered while non-target associative analysis is performed in tandem to provide a deeper more comprehensive approach to raw signal data. This associative analysis is performed by classifying peaks as particular known congener regardless if the peaks are a target or non-target. Through this approach, non-target associated information can be used as a tool to fingerprint sources of analytes. Specifically, associated non-targets discovered and quantified through this method can be used to differentiate between closely related chemical fingerprints of industrial pollutants, e.g. household paints and cleaners that can have almost identical target composition but vary in their distribution of non-target analytes. Another application this algorithm can be directly applied to is petroleum forensics in the aftermath of an oil spill, where the contribution of non-target hydrocarbons to the chemical fingerprint of an oil spill source is typically overlooked.

This paper is divided into five sections. The remainder of this section describes current-state-of-the-art methods and key contributions of this work in applying machine learning techniques to assist in associating target and non-targets found within the signals. Section II provides a brief description of the instrument and raw signal data explored. Section III presents the peak fitting procedures of the signals and Section IV describes the performance metrics to evaluate the machine learning algorithm. Section V discusses the results produced from this proposed technique. Finally, Section VI describes concluding remarks and future work.

### A. CURRENT-STATE-OF-THE-ART

Target-cognizant techniques developed for raw data analysis of gas chromatography-mass spectrometry (GC/MS/MS) data sets have automated analytical interpretation [1]–[7]. The technique developed in Lavner *et al.* uses a peak to noise ratio and intensity comparison to blank peaks which can exclude contributions of lower intensity contributing peaks. Similarly, our previous work uses a thresholding of the raw signal data which ignores smaller peaks presence. PARAFAC implements an N-way principal component analysis (PCA) decomposition that can locate peaks across multiple samples [3]. While PARAFAC can locate and associate peaks with target peaks, it struggles to accommodate for retention time shifts and can overlook smaller contributing peaks. PARAFAC2 fixes the retention time shift issue using a time loading matrix for each sample and uses a similar N-way PCA decomposition to locate peaks but is challenged in determining other reoccurring peaks and identifying the number of optimal components for peak decomposition [4]–[6]. Similar techniques and approaches can also be seen in softwares for GC/MS/MS data as presented in Meyer *et al.* and Wylie *et al.*. While these softwares offer convenient automated analysis, they are prone to misinterpret data due to retention time shifts. Additional injection and hardware approaches have been explored to achieve better extraction of pertinent information [10], [11]. While the developed ideas have proved reliable, they require additional materials that may not be available. Further, injections of particular compounds, as suggested in Kaspar *et al.*, may only be useful for detection of certain chemicals. Recent work in machine learning have introduced new developed analysis of GC/MS/MS and other similar datasets [12]–[16]. While target only approaches such as Skarysz *et al.* can identify well-known contributing analytes in information rich signals, they fall short of associating and identifying non-target contributions. These non-target contributions and associations could provide a more detailed story of source contributions. Though non-targets are not explicitly known and can coelute with targets in raw signals, they could provide more comprehensive information to aid in attenuating sources of contaminants. Alternative approaches to target-cognizant approaches have been developed in recent years [17]–[20]. Target-cognizant approaches such as Domingo-Almenara *et al.* compares non-target low intensity peaks to a larger library of identified non-target compounds across multiple data. An alternative target-cognizant approach presented in Sirén *et al.* and Liigand *et al.*, identifies all peaks in the raw signal and compares the results across multiple samples to identify target peaks within. This approach does not require any a priori information to identify target peaks but is susceptible to smaller data sets where a priori information can aid in target peak association.

### B. KEY CONTRIBUTIONS

This work harnesses pre-processing techniques from recent work presented in McCarthy *et al.* to interface with popular machine learning architectures to provide autonomously annotated signature profiles of different diverse geographic locations that are well known to have PCB contamination [2]. Our previous work analyzes individual MRM signals using signal processing techniques to identify and quantify the relative abundance of a certain congener present within the sample. This was done by first adjusting the signals for potential contamination and correcting for retention time shifts within the signals. Through this, a peak fitting method was implemented to determine the amount of a congener was present in the signal. Finally, the fitted peaks are determined as particular congeners by comparing the retention times of fitted peaks to the known peaks of calibration signals. This work harnesses the pre-processing methods from our previous work through peak alignment correction for retention time shifts that occur. While McCarthy *et al.* annotates target peaks using only unsupervised signal processing techniques, this work annotates and associates target peaks with non-target peaks through a trained machine learning random forest ensemble. Further, this work improves on McCarthy *et al.* by using both a Gaussian peak fitting curve to better quantify the presence of a congener as well as an L2 norm approach as a post-processing algorithm to ensure correct peak association. Through the signal processing techniques in our previous technique, identified peaks within the signal can be used as features for classification. Features in this work are the relative difference in retention time from peaks to identified target standard peaks (i.e. known injected target peaks) as well as which MRM signal the peak is identified in.

**FIGURE 1.**
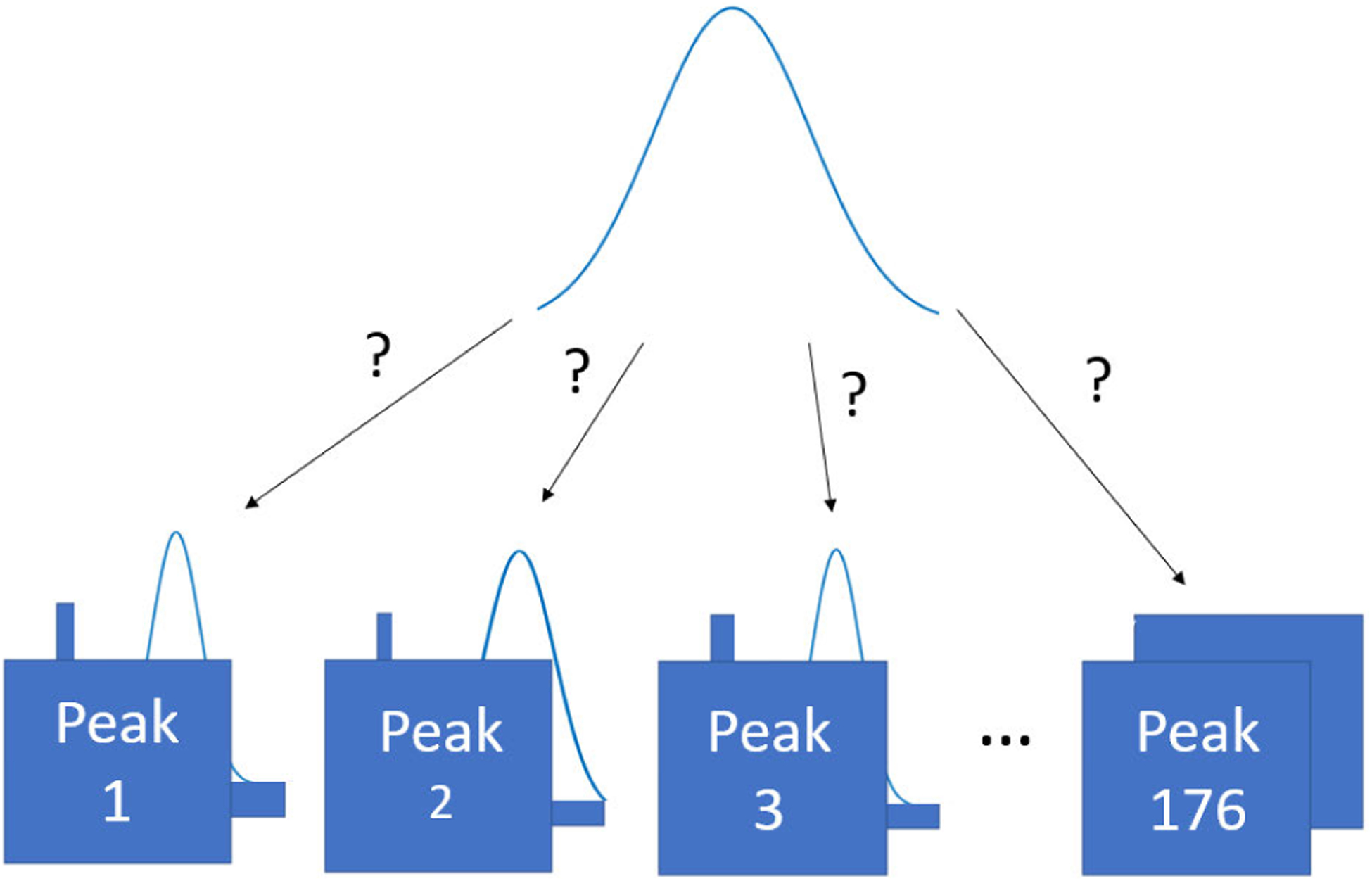
Using the machine learning model, we classify peaks within the signal as one of the 176 different classes (target annotated peaks) that we can identify through the calibration signal. By classifying each peak in the signal, we are associating the peaks with annotated peaks.

The key contribution of this study is to provide a signal processing and machine learning approach to identify target polychlorinated biphenyls (PCB) within GC/MS/MS signals autonomously and associate potential non-target contaminants found. Specifically, this work analyzes the performance of a random forest, support vector machine (SVM), and naive Bayes classifiers to autonomously identify target PCB peaks and evaluates the performance through several statistical equations. This machine learning approach can be visualized as classifying individual peaks as a one of the known target classes (seen in Figure 1). Each target class represents one of the 176 specifically known PCB congener. Because of coelution of particular PCBs within the MRM signals, manual analysis of the raw signal is limited from the 209 known classes. While there are 209 known PCB congeners, 176 congeners are used for machine learning because these congeners were manually analyzed within the provided data. Further, this work examines location specific samples to build signal geographical templates to help fingerprint and examine PCB topography in regions. Geographical templates demonstrate the average contributions of known target PCBs present within a region which can be used to help track increases of PCBs within a region.

## II. DESCRIPTION OF DATA

Gas chromatography mass spectrometry (GC/MS) has helped identify analytes in samples by separating the gases as they move through the column. As compounds separate, the detector records the column at different speeds and produces a chromatogram with the peaks corresponding to concentration amounts of different chemicals [21]–[23]. Although this instrument provides powerful analysis of the samples, it can be prone to complications such as retention time shifts, column bleeding, and coelution of peaks in the chromatogram. To increase sensitivity of the machine, a tandem mass spectrometer (MS/MS) was made which has two scanning mass analyzers that are separated by a collision coil [24]. This works by taking the fragments from the first analyzer and putting it through an inert gas within the collision cell thus separating the compounds further. Using this in tandem with multiple reaction monitoring (MRM), mass transition states are created to determine trace amounts of compounds in the samples while filtering out other contaminants [25]. While the capability to separate compounds from other contaminants is plausible, automatic interpretation of the data can be difficult due to coelution, retention time shifts, and potential co-occurring contaminants that may have similar masses.

The MRM signals provide specifically filtered samples that decompose TIC signals to homolog specific groups enhancing quantification of contamination. Each MRM signal, *H*[*n*], is a linear summation that contributes to the total TIC signal, *T* [*n*]. Mathematically this is calculated as

(1) $$T[n]=\overset{M}{\underset{i}{\sum }}H_{i}[n]$$

**FIGURE 2.**
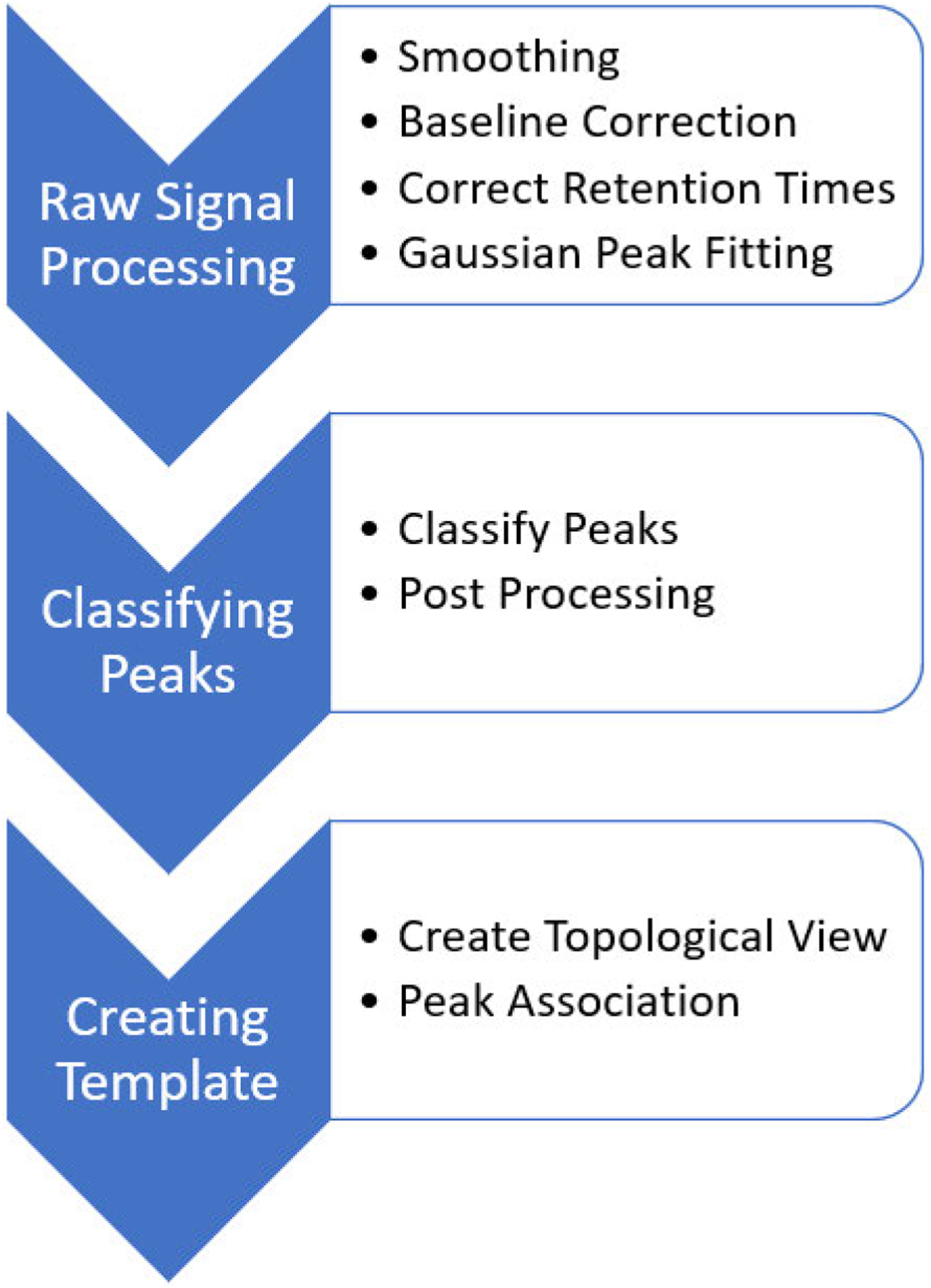
Procedure to identify annotated and non-target peaks within the signals.

While MRM signals are highly selective and enhance statistical analysis of pollutants, empirically examining every signal is time consuming and biased toward known target contaminants. To ensure consistent, accurate, and higher dimensional analysis of the MRM signals, a suite of signal processing techniques and machine learning techniques are implemented to autonomously evaluate the signals. This work provides a detailed procedure of pre-processing, machine learning, and post-processing techniques to analyze the MRM signals. The process to decompose the data is presented sequentially within the next section and the outline of this process can be seen in Figure 2.

## III. TECHNICAL APPROACH

### A. PRE-PROCESSING FOR MRM SIGNAL CORRECTION THROUGH SIGNAL PROCESSING

#### 1) SMOOTHING RAW SIGNAL CHROMATOGRAPHY

**FIGURE 3.**
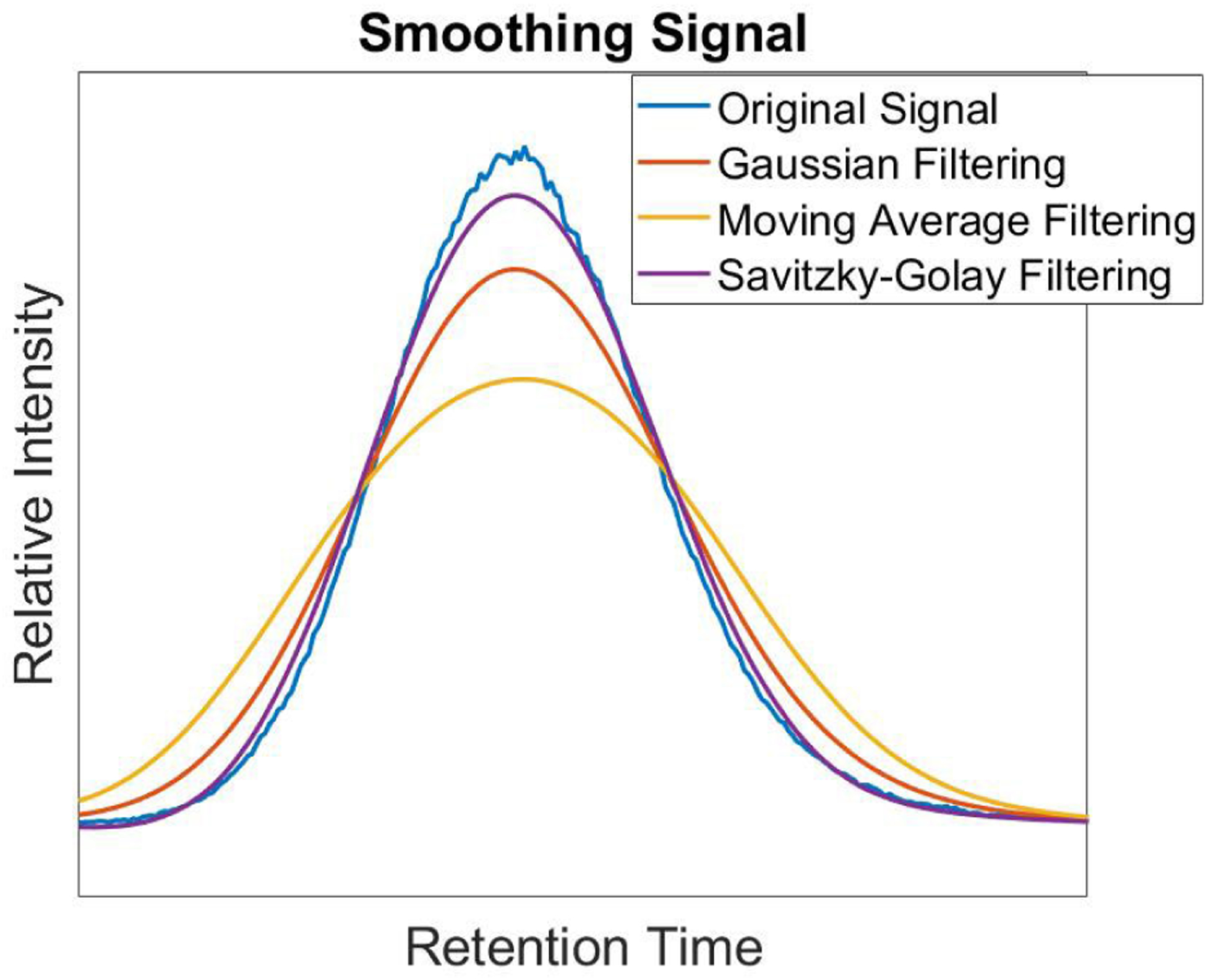
Smoothing an MRM signal using various filtering techniques.

Instrument noise can cause raw MRM signals to have miscellaneous peaks impacting the analysis of the data. To ensure sufficient signal analysis, noise is removed through smoothing. In this work, noise is removed by utilizing a Savitzky-Golay filter. A Savitzky-Golay filter is used because it is effective at preserving larger peaks within the signal and less likely to reject smaller potentially target peaks therein [26], [27]. An example of this smoothing can be seen in Figure 3 where the Savitzky-Golay filter is compared to a Gaussian filter and moving average filter. The new windowed filtered sampled signal, *Ĥ*[*n*], is calculated using:

(2) $${\hat{H}}_{i}[n]=\overset{N}{\underset{j=0}{\sum }}\alpha_{k}m^{k};-d\leq m\leq d$$

where *α*_*k*_ is the *k*^*th*^ coefficient, *d* is the half-width of the samples (i.e. 2*d* + 1 is the full length of samples) and *N* is the polynomial order. We calculate *α* as:

(3) $$\alpha =A{\left(A^{T}A\right)}^{-1}A^{T}h$$

where *h* is a (2*d* + 1) × 1 column vector impulse, i.e. a section of the signal *H*_*i*_[*n*] to be smoothed, and *A* is:

(4) $$A=\left[\begin{array}{cccc} {\left(-M\right)}^{0} & {\left(-M\right)}^{1} & \ldots & {\left(-M\right)}^{N}\\ \vdots & \vdots & \ddots & \vdots \\ {\left(M\right)}^{0} & {\left(M\right)}^{1} & \ldots & {\left(M\right)}^{N} \end{array}\right]$$

where the size of *A* is (*N* + 1) × (2*m* + 1). This equation is explored further in Schafer.

#### 2) ELIMINATING ADDITIONAL NOISE AND EXTRA CONTAMINANTS BY ADJUSTING CHROMATOGRAPHY BASELINE

In some instances, the instrument will have column bleeding which is the background signal generated by column stationary phase. This will cause the signal to elevate over time making it appear as though there is more contamination than there is in addition to making the information harder to quantitate. Another issue that can occur is when there is column contamination from material leaking through to the detector. Due to these issues, it can be difficult to quantify how much of a substance is present in a sample. To fix this, a shape-preserving piecewise cubic interpolation regression is implemented with a window size of 400 and a quantile value of 0.05 to regress the signal to a better baseline. While other techniques such as linear interpolation and spline interpolation can be used for regression, the shape-preserving piecewise cubic interpolation provides a softer regression and optimizes to return positive intensity values. This shape-preserving piecewise cubic interpolation regression is calculated between the intervals [*n*_1_*, n*_2_] as:

(5) $$s_{i}[n]=a{\left(n-n_{i}\right)}^{3}+b{\left(n-n_{i}\right)}^{2}+c\left(n-n_{i}\right)+d$$

where *s* is the new baseline and [*abcd*] are the cubic interpolation coefficients. The cubic interpolation coefficients are calculated through derivation and explained further in de Boor. The new MRM signals are readjusted as:

(6) $${\tilde{H}}_{i}[n]={\hat{H}}_{i}[n]-s_{i}[n]$$

#### 3) ADJUSTING RAW SIGNAL RETENTION TIME DRIFTS

**FIGURE 4.**
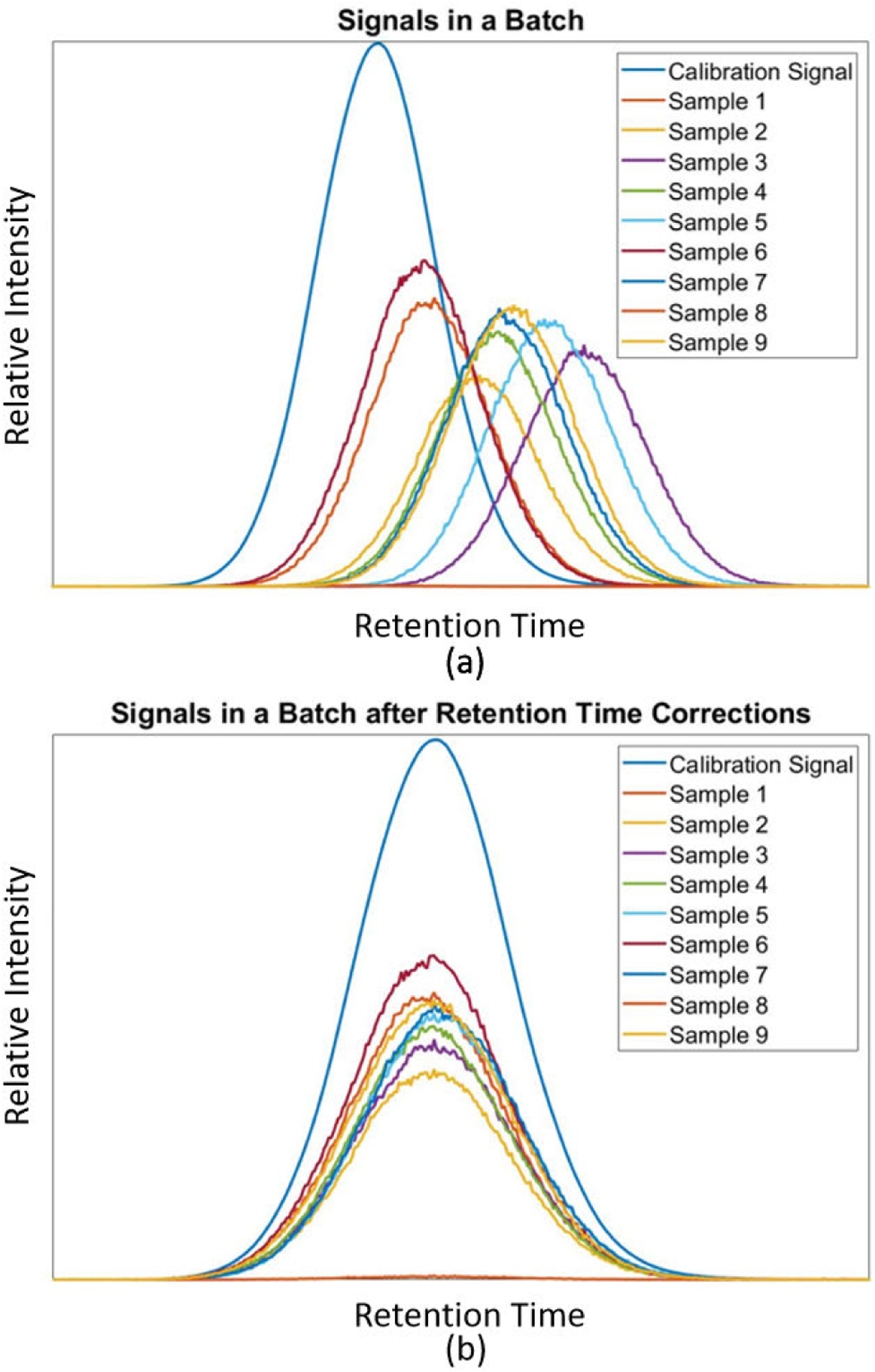
Example of a batch of samples and the retention time drift that occurs in the signal.

**FIGURE 5.**
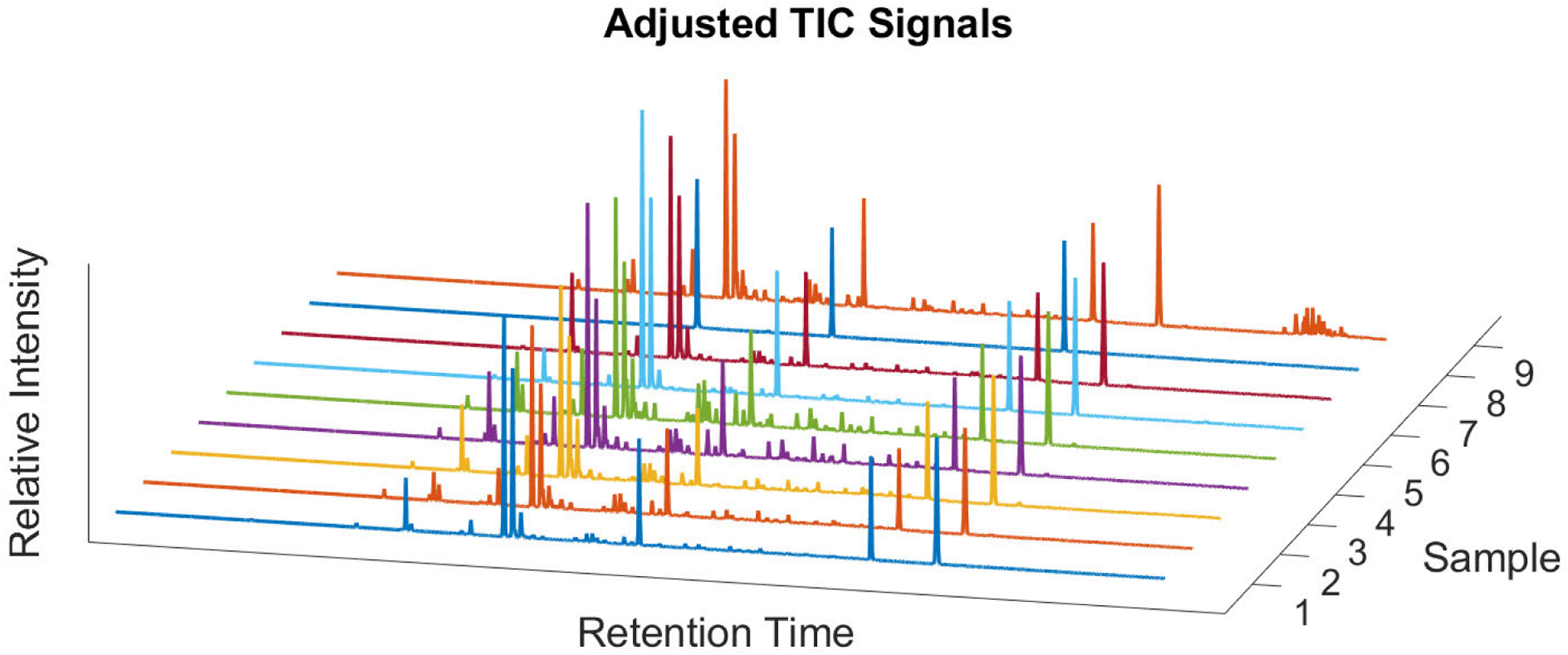
A batch of samples that has been processed and adjusted to take out extra contaminants, fix retention time drifts, and be uniform across the samples.

The MRM signal drifts from sample to sample as a result of changes in column temperature, dimension, or linear velocity of carrier gas through the columns [29]. Seen in Figure 4 (a) is an example of the retention time shift that occurs in the sample data sets. Due to the complexity of the signals, it is difficult to autonomously determine peaks correspondence to the calibration signal peaks. To correct for these shifts, standards (high relative intensity peaks) are injected within samples before runs and are used within the calibration signal to align MRM signals correctly, similar to the technique in our previous method. MRM signals are corrected by aligning the standard that is associated with the MRM signal to the standard within the calibration signal. Shifting the standard peaks in each sample to align with the calibration standard peak, the MRM signals are realigned to have consistency within the signals (as seen in Figure 4 (b)). Figure 5 demonstrates a group of TIC signals after being pre-processed.

#### 4) FITTING PEAKS WITHIN CHROMATOGRAPHY TO DETERMINE COELUTION

**FIGURE 6.**
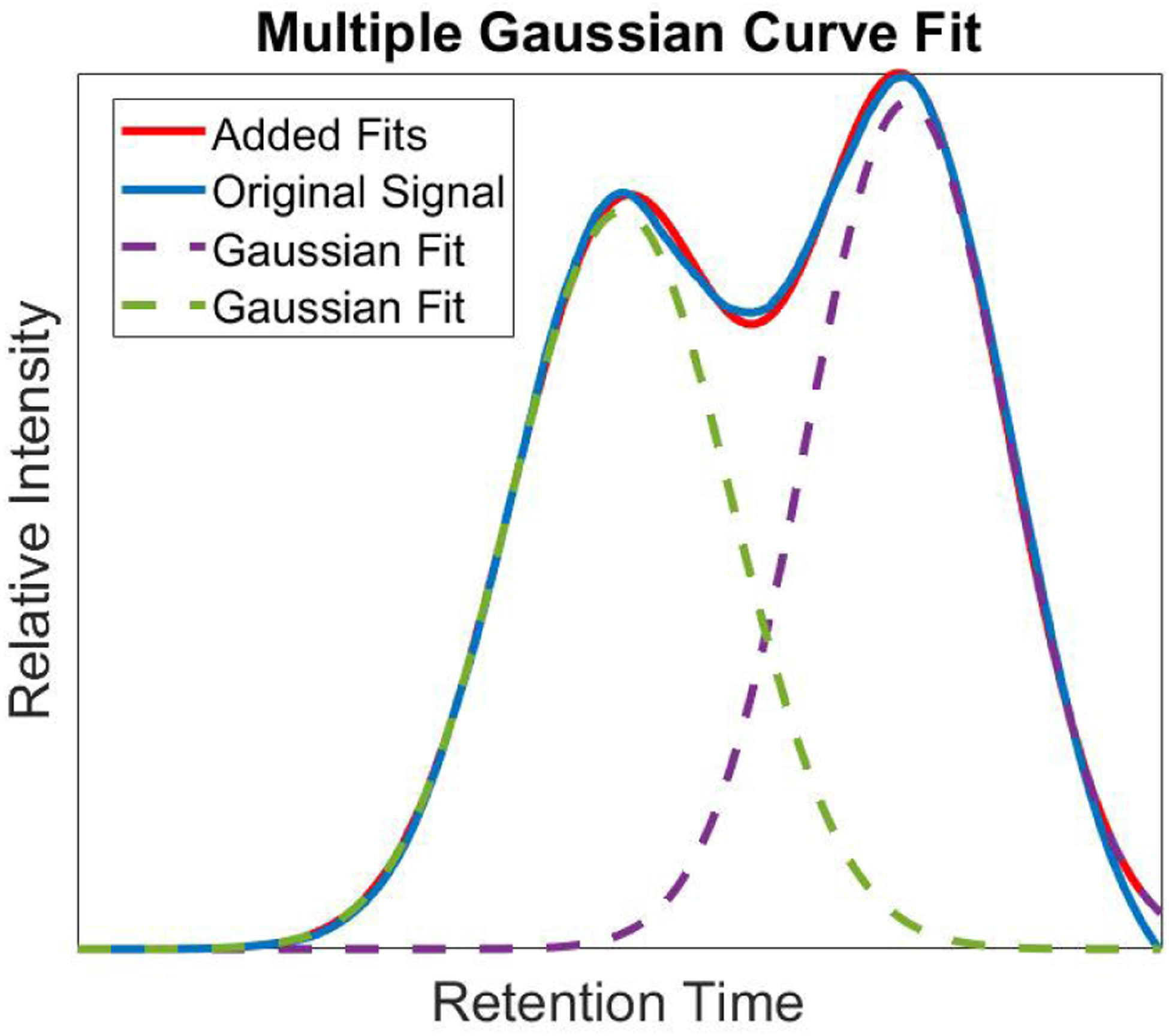
Multiple Gaussian curves fitted to a coeluted peak providing better analysis of total amount of a PCB congener within the sample.

To analyze the available MRM raw signals for different analyte groups, peak fitting is employed to autonomously determine the contribution of coeluted and non-coeluted peaks within the signal. To derive the individual peak heights, which correspond to pertinent congeners within the samples, each peak is fitted using MATLAB’s Gaussian fit function. While the cosine peak fitting algorithm presented in our previous method demonstrated a superior fit for individual contributions of peaks, the Gaussian fit function is able to decompose the peak into distinct contributing peaks with an overall goodness of fit above 90%. The number of peaks determined to fit the peak are first chosen by first comparing the number of peaks that are close in retention time to the calibration signal. Once fit, the number of peaks to fit the curve are adjusted iteratively until a goodness of fit is above 90%. Mathematically, the Gaussian peak, *y*, total contribution can be seen as:

(7) $$y=\overset{N}{\underset{i=1}{\sum }}\beta_{i}\;\text{exp}\left(-\frac{n-B_{i}{}^{2}}{\sigma_{i}}\right)$$

where *N* is the number of peaks to fit, *β* is the amplitude coefficients, *σ* is the peak width, and *B* is the centroid location. The coefficients and solutions for the peak contributions are optimized through a trust region as discussed in Moré *et al.*. Further implementation and documentation can be seen in MATLAB’s *Curve Fitting Toolbox User’s Guide*. An example of the Gaussian fit and capability to deconvolve a coeluted peak can be seen in Figure 6.

### B. MACHINE LEARNING

Three machine learning algorithms are considered: random forest, support vector machine (SVM), and naive Bayes. Each algorithm is trained, implemented and evaluated in different metrics to pick the best algorithm to use. These three algorithms were chosen due to their unique approaches to classifying data given certain features. Each machine learning algorithm has four inputted features. The four features are the difference in retention time from three of the injected standards as well as which MRM the peak belongs to. The data is partitioned to have 80% of the sample data as training and 20% of the data as testing. The 20% of data determined for testing were from location specific data to do further analysis. The model is validated using a 7-fold cross validation of the data. The output of the algorithm is to determine which peak class to associate. Classes are assigned as one of 176 PCB values where each class number corresponds to a specific target congener identified within the calibration signal (e.g. value 1 corresponds to target PCB 1).

### C. POST-PROCESSING

Once peaks within the signal are classified through machine learning, the peak within the class that matches closely to the calibration annotated peak will be designated as the target peak. To assign the peak, a L2 norm is utilized. This is done by choosing the peak within the class of peaks, *C*, that has the largest height, peak area, and is relatively close in retention time to the calibration annotated peak. Mathematically the best choice peak for each class, *G*, is seen as:

(8) $$G=\underset{i\in C}{\text{max}}{\left\|\Theta_{i}^{2}+p_{i}^{2}+{\left(\frac{1}{t_{i}-r_{j}}\right)}^{2}\right\|}^{1/2}$$

where Θ is the peak height, *p* is the peak area, *t* is the retention time of the classified peak, and the *r* is the retention time of the calibration annotated peak. Equation 8 prioritizes the relative relationship in retention time between the classified peaks and annotated calibration peak because of the pre-processing techniques implemented and discussed earlier. When peaks are highly coeluted within the signal, e.g. fitted peaks that have similar retention time differences from the known target peak, Equation 8 determines the best peak by assessing both the peaks height and area.

## IV. PERFORMANCE METRICS OF MACHINE LEARNING ALGORITHMS

This work evaluates each machine learning algorithm ability to autonomously identify target PCB peaks based on accuracy in training and testing as well as the precision, recall, and F1 score of testing data. Ground truths to determine these metrics are provided and documented in Hu *et al.*. Moreover, ground truths for each of the annotated peaks are considered based on existing calibration standards manually examined. Additionally, manually executed expert validation for each sample was performed documenting whether a peak was identified and present at the same retention time as the annotated calibration signal. PCBs may or may not be present in the raw signal due to low concentrations of such PCBs in samples. Therefore, there are four possible labels which can be assigned for each PCB:

TP: A PCB peak in the sample is found manually and autonomously as an identified peak in the calibration signal;
TN: A PCB peak in the sample is not found manually and autonomously as an identified in the calibration signal;
FP: A peak in the sample is found autonomously and is labeled as a PCB peak identified in the calibration signal but the manual inspection does not detect such peak;
FN: A peak in the sample is found manually and is labeled as a PCB peak identified in the calibration signal but the autonomous inspection does not detect such peak;

The metrics are significant in determining a model that will correctly identify and classify peaks in the signals without misrepresenting the data. Using the above metrics, accuracy can be mathematically expressed as:

(9) $$\text{Accuracy}=\frac{TP+TN}{TP+TN+FP+FN}$$

While accuracy alone can indicate a sufficient model, the results could be skewed in its classification. In such case, the model could have difficulty in reporting any PCB peaks or even incorrectly classifying peaks. More importantly, the results from the machine learning models must not falsely classify peaks as present or not. To address this, the F1 score is calculated which measures the precision and recall of the model. Through the F1 score, a better measure of incorrectly classified peaks is determined. Precision measures all the correctly identified positive cases from all the predicted positive cases as:

(10) $$P=\frac{TP}{TP+FP}$$

Precision shows how well autonomously annotated peaks identified are present compared to the manually identified peaks. Similarly, recall measures the correctly identified positive cases from all the actual positive cases as:

(11) $$R=\frac{TP}{TP+FN}$$

Recall calculates the number of instances that manual and autonomous detection of annotated peaks aligned and accounts for failure of peak association in autonomous classification. Using both Equation 10 and Equation 11, the F1 score is calculated as:

(12) $$F1=2\left(\frac{P*R}{P+R}\right)$$

The F1 scores conveys how well the algorithm balances precision and recall when predicting classifications. Further, the F1 score is evaluated in this work because there is an imbalance in class distribution (i.e. there are mainly annotated peaks present in the data) therefore it is important to understand the performance of recall and precision of the machine learning algorithms. The results of each machine learning algorithm is discussed in the next section and the best machine learning algorithm is chosen to perform deeper analysis.

## V. RESULTS AND DISCUSSION OF SIGNAL PROCESSING TECHNIQUES FOR RAW SIGNAL ANALYSIS OF ENVIRONMENTAL POLLUTANTS

High-volume air samplers were deployed across the Chicago metropolitan area from 2007 to 2009 to get 150 collective air samples to analyze PCBs [32]. Samples were analyzed using a GC-MS/MS (Agilent 7000 Triple Quad with Agilent 7890A GC and Agilent 7693 autosampler equipped with a Supelco SPB-Octyl capillary column) in MRM mode [33], [34]. Surrogate standards recoveries, replicates, laboratory and field blanks, and standard reference material were included for analytical quality control. Twelve chromatograms were produced through MRM for each sample. Each chromatogram represents the chromatographic signal for different mass transition ions (10 transitions for unlabeled PCBs and 2 for mass-labeled PCB standards). The MRM mass transitions used can be seen in Table 2. Further, one TIC signal was obtained for each sample, representing the linearly accumulated MRM signals. A MRM and TIC signal were obtained for the calibration solutions containing 209 PCB congeners. Each peak within the TIC and MRM signal should correspond to a PCB congener or a non-target chemical found in the sample. Relative intensities were used as the total amount of the PCB congener or chemical found. MRM and TIC chromatographic signals were first manually adjusted for appropriate and consistent baseline using Agilent’s software MassHunter (Version B.06.00, ©Agilent Technologies, Inc). The algorithms and further analysis was performed using MATLAB R2018a software (The Mathworks, Inc. USA). The toolboxes: Bioinformatics toolbox, Optimization toolbox, and Statistics and Machine Learning toolbox were installed to implement the algorithms. Further details can be seen in McCarthy *et al.* and Hu *et al.*.

The resulting analysis of the Chicago air data was performed with a random forest algorithm on the testing data to investigate and develop templates of location specific samples. The random forest algorithm was chosen because it had the highest F1 score and testing accuracy as compared to the SVM, and naive Bayes (seen in Table 1). All annotated peaks were determined using Equation 8 for each sample and aligned to show how annotated peaks accumulate and degrade in time. Four sampling locations are considered for analysis: Addams, Metcalfe, St. Sabina, and Rey Gonzales which are spread out across the Chicago area. All samples were pre-processed, annotated, and post processed using Section III. Further, templates and non-target analysis were assessed for each location and results are presented next. The trained machine learning algorithm can be adapted to other data sets by picking out similar MRM mass transition groups as seen in Table 2 when classifying peaks.

### A. ANNOTATED TARGET IDENTIFIED PEAKS ACROSS SAMPLES

**FIGURE 7.**
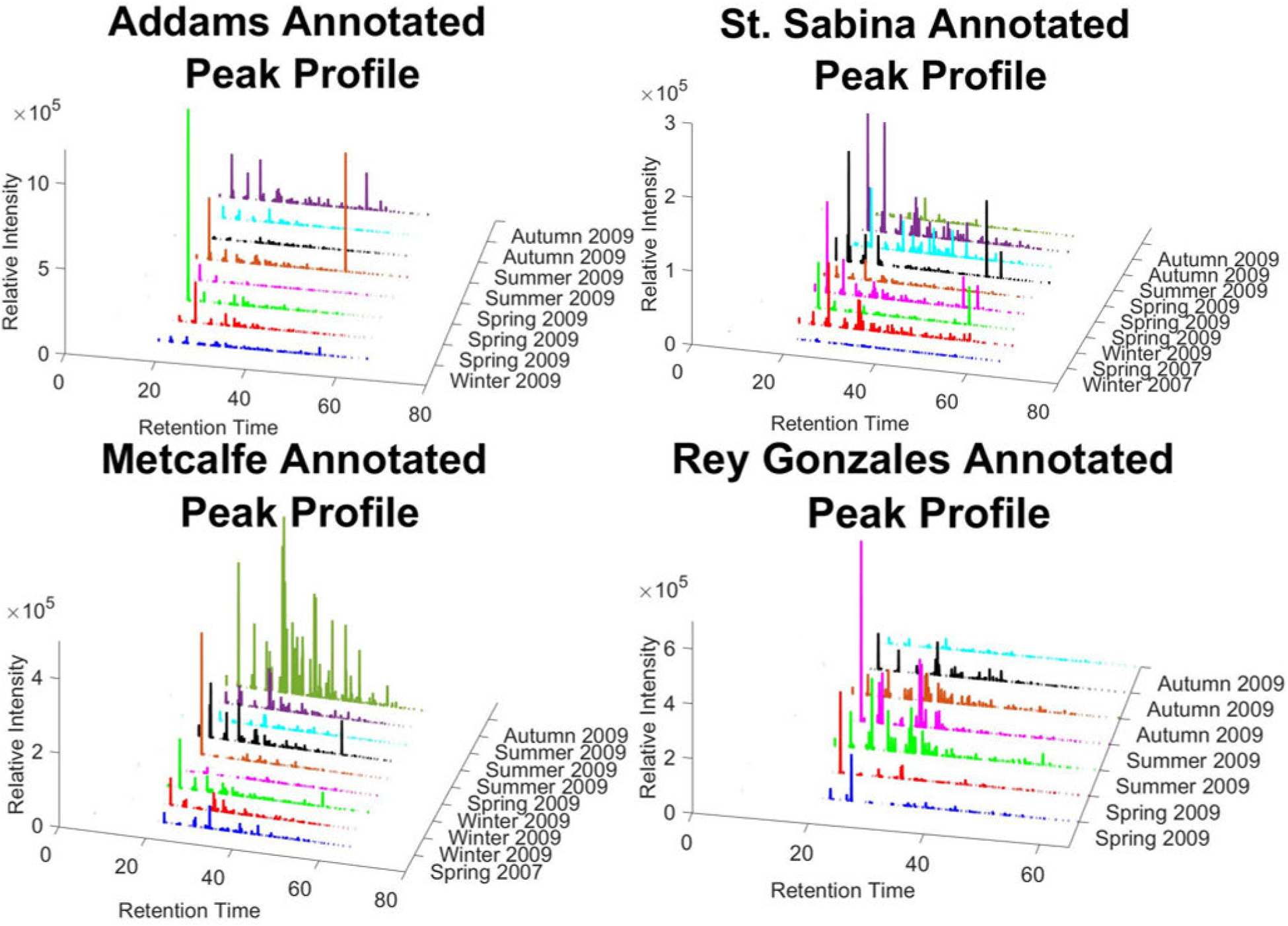
Topographical demonstration of autonomously annotated peaks achieved using machine cognition presented in this work across multiple geographical sampling locations that are well known to have PCB contamination. Each sampling location has samples across different years and seasons. Each sampling location signal is aligned and dates are sequentially ordered to determine PCB congener contributions.

Analyzing each sample individually, similar specific geographical sampling locations were extracted and aligned. Figure 7 demonstrates samples of individual locations across the years. The annotated peaks identified exclude the injected standards that bias the overall relative intensity. Concentrations of PCBs seen in Metcalfe increase across time and relative intensity while relative intensity of PCBs in Rey Gonzales slightly decreases over time. This technique allows for more intuitive analysis of location specific sampling and can provide better chemometric analysis. Using random forest classification on larger data sets and applying sample signal comparison as seen in 7 can identify PCBs that may accumulate or degrade over time. While this work explores target annotated peaks within the GC/MS/MS MRM data sets, it can also be used for non-target peaks within location specific samples demonstrated in Section V-C. The templates created in the next section are created by linearly adding all annotated peaks and averaging the relative intensities by the number of samples.

### B. LOCATION SPECIFIC SIGNAL TEMPLATE

**FIGURE 8.**
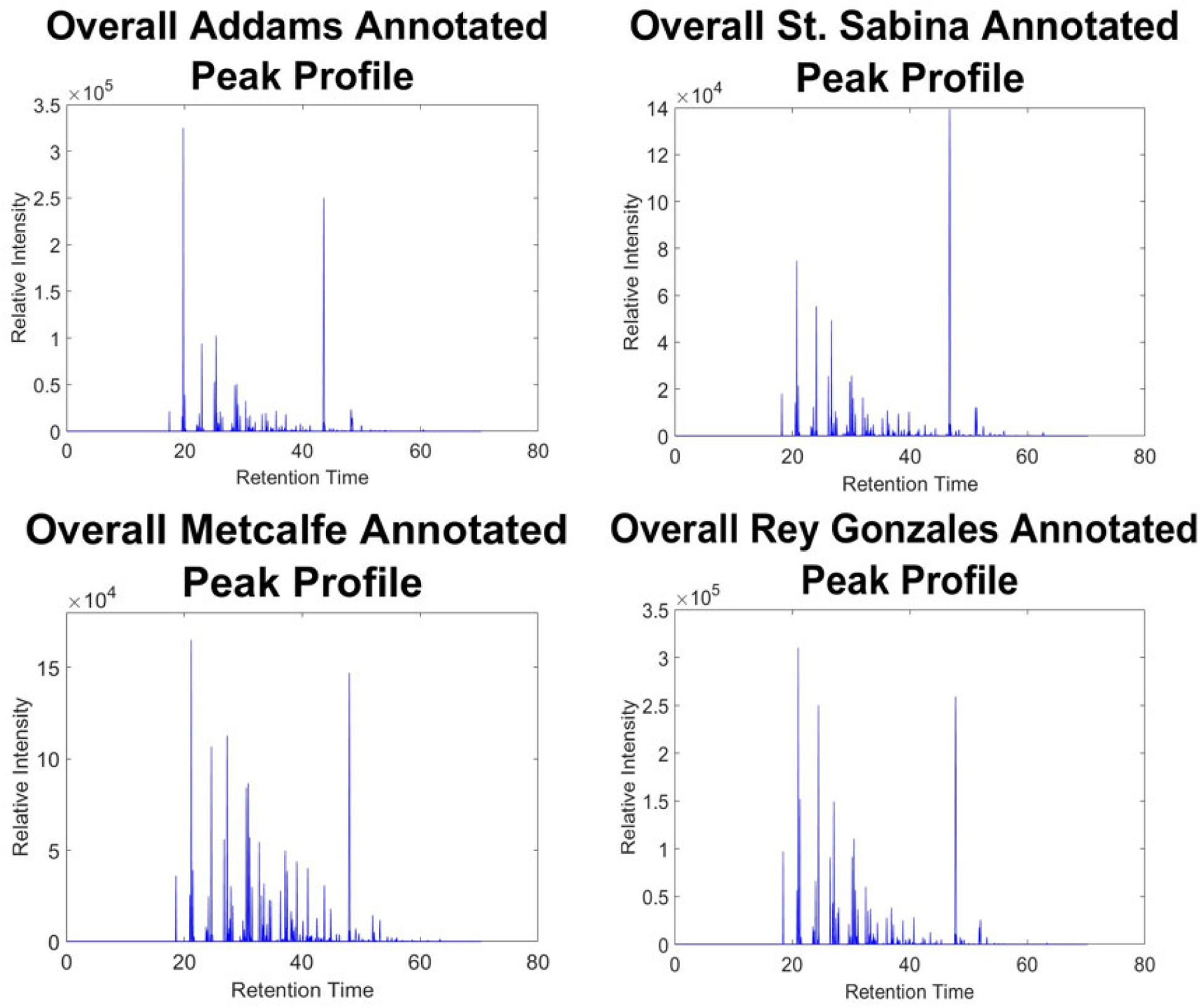
Templates of overall autonomously annotated peaks achieved using machine cognition presented in this work across multiple geographical sampling locations that are well known to have PCB contamination. Each template is developed by a linear summation of annotated peaks seen in Figure 7.

Leaving out the few location specific samples from our machine learning, we examined the signals annotated target PCB peaks. The samples that we examine are spread out across the years and the seasons so to better understand the sample location. Seen in Figure 8 are the annotated peaks identified from the procedures discussed in Section III. From these different templates it can be seen how the signal varies from year to year and season to season providing a picture of variability. Something to note is that the signal relative intensity differs across the different locations and even the samples themselves. This could be for a variety of reasons one of which could be the season that the sample was collected. These contaminants are more prone to release into the air during the warmer time of the years (i.e. Summer or Spring) thus we see a higher relative intensity of annotated peaks from these samples. Another reason that these samples have higher relative intensities are based on the sampling location itself. As seen in Figure 8, the majority of annotated peaks at St. Sabina are smaller than those at Metcalfe or Rey Gonzales. This difference in relative intensity could be because of how close the sampling device is to a source of PCBs.

### C. NON-TARGET PEAKS CONSISTENTLY FOUND

Through the machine learning technique we developed earlier, we note that we have classified the peaks in the signal as specific annotated peaks (as demonstrated earlier in Figure 1). This is important for association and determining non-target peaks because peaks that consistently get associated with annotated peaks and show up consistently across samples could be considered a peak that may be of interest (a potential contaminant that is showing up with one of the annotated peaks). This is useful for source identification since peaks that are not annotated peaks, may have extra information about the source that is causing the contamination in the location.

**FIGURE 9.**
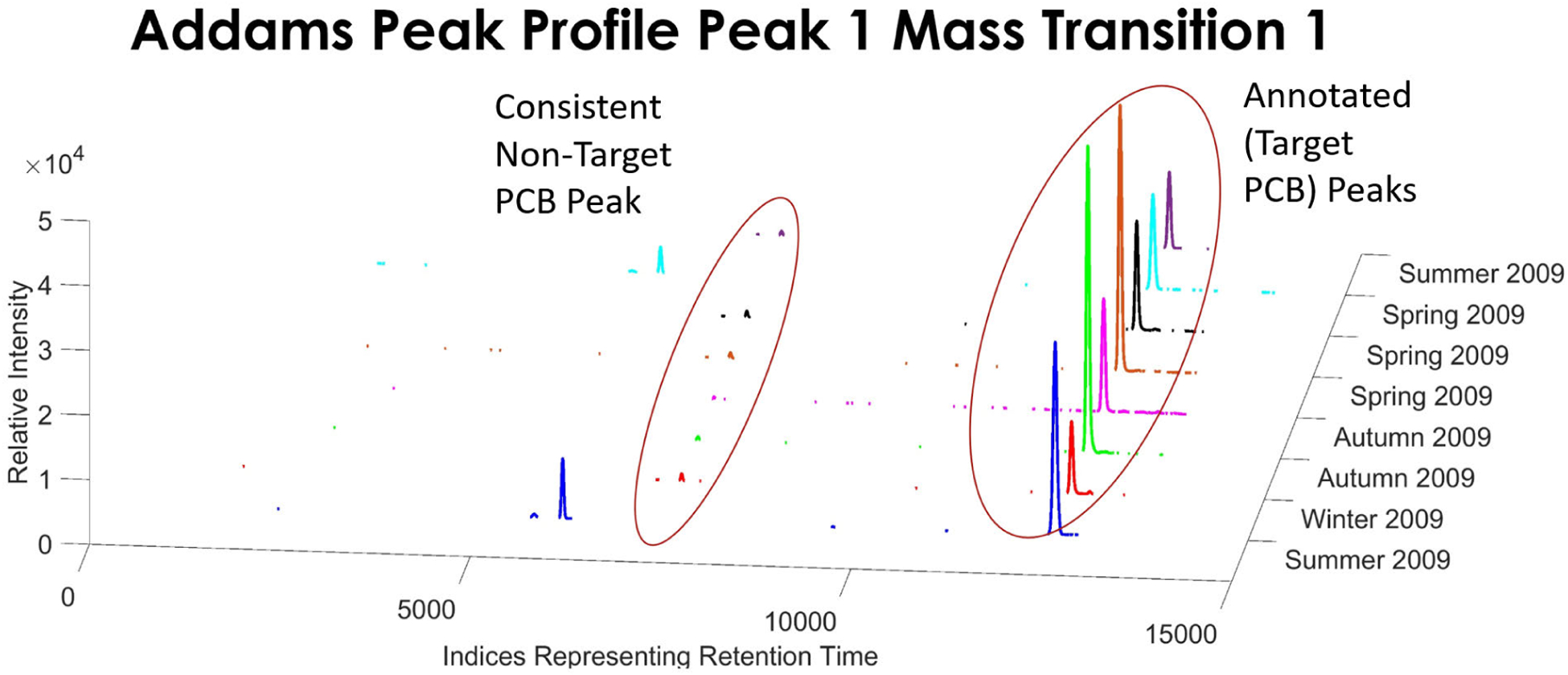
Example of the non-target peak found in the first mass transition signal which is associated with the first peak of the annotated peaks.

Examining the first annotated peak in the first mass transition signal, we identified a peak that was consistently showing up in the signals, around the same retention time, and across nearly all the locations we have examined (seen in Figure 9). Although this peak is small and not seen in every sample, it’s still consistent and gets classified with the first annotated peak that we are able to identify successfully.

## VI. CONCLUSION

This work proposes a sequential combination of various computational techniques to automate the detection and interpretation of peaks found within GC/MS/MS data sets. Specifically, a robust pre-processing procedure is employed trough signal processing to separate and identify peaks within the MRM data. Then, machine learning is employed to group peaks within the raw data as potential PCB congeners associating target and non-target peaks through classification. Through our post-processing criterion, individual peaks within the groups are isolated as the contributing PCB congener. These annotated peaks are demonstrated across four sampling locations that represent a diverse portfolio of Chicago air. Utilizing these techniques within the GC/MS/MS signal can provide beneficial chemometric analysis that is overlooked in individual sample analysis. This comprehensive technique is valuable to GC/MS/MS analysis in three significant ways:

- Robust pre-processing technique eliminates additional noise and contamination of non-significant peaks within the GC/MS/MS data providing comprehensive analysis of raw signal data.
- Machine learning classification through peak based features provide associative clustering techniques to interpret large-scale analysis at the level of individual compounds.
- Comprehensive templates and location specific sample evaluation to distinguish PCB congener degradation, or accumulation, across time. This approach can distinguish particular re-occuring non-target peaks hidden within the signals.

The major scientific return of our techniques is using machine learning as a automated clustering and classifying technique to connect target compounds (known PCB congeners) with potentially significant but previously unknown non-target compounds. Further, this technique analyzes signal topography to identify peak contributions across time and annotate non-target peaks. In this work, we report our findings across 150 active air samples, but this technique and procedures can be applied across much larger scales of data and repositories.

## Figures and Tables

**TABLE 1.** Accuracy of the training data is shown in the top of the table. Accuracy, precision, recall, and F1 score of the training annotated peaks of the test data is shown in the bottom part. The random forest classification algorithm performed better than the SVM and Naive Bayes classification algorithms.

Training Data			
	Random Forest	SVM	Naive Bayes
Accuracy	99.2%	99.2%	95.5%
Testing Data			
	Random Forest	SVM	Naive Bayes
Accuracy	85.28%	84.97%	78.39%
Precision	98.07%	98.06%	98.22%
Recall	86.68%	86.36%	79.39%
F1 Score	92.02%	91.84%	87.81%

**TABLE 2.** MRM Mass Transitions (M/Z) of data used.

Cl Homolog	Precursor Ion	Product Ion
1	188.0	152.0
2	222.0	152.0
3	258.0	186.0
4	291.9	222.0
5	325.0	255.9
6	359.8	289.9
7	393.8	323.9
8	429.7	259.8
9	463.7	393.8
10	497.7	427.9
